# Abnormal regulation of microRNAs and related genes in pediatric β‐thalassemia

**DOI:** 10.1002/jcla.23945

**Published:** 2021-08-16

**Authors:** Haiwei Wang, Meihuan Chen, Shiyi Xu, Yali Pan, Yanhong Zhang, Hailong Huang, Liangpu Xu

**Affiliations:** ^1^ Medical Genetic Diagnosis and Therapy Center Fujian Maternity and Child Health Hospital Affiliated Hospital of Fujian Medical University Fuzhou China; ^2^ Guangxi Medical University Nanning China; ^3^ Medical Technology and Engineering College of Fujian Medical University Fuzhou China; ^4^ Fujian University of Traditional Chinese Medicine Fuzhou China

**Keywords:** B‐cell lymphoma/leukemia 11A, *let7* microRNA, microRNA sequencing, pediatric β‐thalassemia, γ‐globin reactivation

## Abstract

**Background:**

MicroRNAs (miRNAs) participate in the reactivation of γ‐globin expression in β‐thalassemia. However, the miRNA transcriptional profiles of pediatric β‐thalassemia remain unclear. Accordingly, in this study, we assessed miRNA expression in pediatric patients with β‐thalassemia.

**Methods:**

Differentially expressed miRNAs in pediatric patients with β‐thalassemia were determined using microRNA sequencing.

**Results:**

*Hsa‐miR‐483‐3p*, *hsa‐let‐7f‐1‐3p*, *hsa‐let‐7a‐3p*, *hsa‐miR‐543*, *hsa‐miR‐433‐3p*, *hsa‐miR‐4435*, *hsa‐miR‐329‐3p*, *hsa‐miR‐92b‐5p*, *hsa‐miR‐6747‐3p* and *hsa‐miR‐495‐3p* were significantly upregulated, whereas *hsa‐miR‐4508*, *hsa‐miR‐20a‐5p*, *hsa‐let‐7b‐5p*, *hsa‐miR‐93‐5p*, *hsa‐let‐7i‐5p*, *hsa‐miR‐6501‐5p*, *hsa‐miR‐221‐3p*, *hsa‐let‐7g‐5p*, *hsa‐miR‐106a‐5p*, *and hsa‐miR‐17‐5p* were significantly downregulated in pediatric patients with β‐thalassemia. After integrating our data with a previously published dataset, we found that *hsa‐let‐7b‐5p* and *hsa‐let‐7i‐5p* expression levels were also lower in adolescent or adult patients with β‐thalassemia. The predicted target genes of *hsa‐let‐7b‐5p* and *hsa‐let‐7i‐5p* were associated with the transforming growth factor β receptor, phosphatidylinositol 3‐kinase/AKT, FoxO, Hippo, and mitogen‐activated protein kinase signaling pathways. We also identified 12 target genes of *hsa‐let‐7a‐3p* and *hsa‐let‐7f‐1‐3p* and 21 target genes of *hsa‐let‐7a‐3p* and *hsa‐let‐7f‐1‐3p*, which were differentially expressed in patients with β‐thalassemia. Finally, we found that *hsa‐miR‐190‐5p* and *hsa‐miR‐1278‐5p* may regulate hemoglobin switching by modulation of the B‐cell lymphoma/leukemia 11A gene.

**Conclusion:**

The results of the study show that several microRNAs are dysregulated in pediatric β‐thalassemia. Further, the results also indicate toward a critical role of let7 miRNAs in the pathogenesis of pediatric β‐thalassemia, which needs to be investigated further.

## Graphical abstract

We performed microRNA sequencing to identify the microRNA expression profiling of pediatric β‐thalassemia. Totally, 530 microRNAs were identified. Based on criteria of fold change >1.5 and *p*‐value <0.05, 111 microRNAs were upregulated in β‐thalassemia patients, while 85 microRNAs were downregulated in β‐thalassemia patients. Those microRNAs could clearly distinguish the normal cohorts from the β‐thalassemia patients. Hsa‐miR‐2100‐3p, hsa‐microRNA‐15a‐5p, hsa‐microRNA‐16‐5p, and hsa‐miR‐503‐5p were all downregulated in pediatric β‐thalassemia patients. We also found five let7 microRNAs hsa‐let‐7b‐5p, hsa‐let‐7i‐5p, hsa‐let‐7f‐5p, hsa‐let‐7e‐5p, and hsa‐let‐7d‐5p and were downregulated in pediatric thalassemia patients.

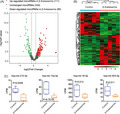

## 1 INTRODUCTION

β‐Thalassemia is one of the most common genetic disorders of blood.^1^, ^2^ There are three subtypes of β‐thalassemia, that is, β‐thalassemia minor, intermedia, and major.^3^ β‐Thalassemia minor is caused by a deficiency in one β‐globin gene, and patients usually have no symptoms or suffer from mild anemia. By contrast, β‐thalassemia intermedia or β‐thalassemia major is caused by double heterozygotes or homozygotes of the β‐globin gene.^4^, ^5^ Patients with β‐thalassemia have major fetal health issues at birth and require lifelong blood transfusions and iron chelation treatments.^6^, ^7^, ^8^ However, current treatments for β‐thalassemia major are associated with severe side effects,^9^, ^10^ and alternative therapeutic approaches are still being developed.^11^, ^12^, ^13^ Therefore, strategies for the prenatal diagnosis of β‐thalassemia are urgently needed, particularly in regions with a high prevalence of β‐thalassemia.

In human developmental processes, β‐like hemoglobin is switched from fetal γ‐globin to adult β‐globin at the time of birth.^14^, ^15^ The absence of or reduction in β‐globin in β‐thalassemia may reactivate γ‐globin expression and fetal hemoglobin (HbF) synthesis.^16^, ^17^ Understanding the molecular mechanisms of the reactivation of fetal γ‐globin expression in adult erythroid cells will provide novel therapies for patients with β‐thalassemia.^18^ B‐cell lymphoma/leukemia 11A (BCL11A) is a major suppressor of γ‐globin^19^, ^20^, ^21^ and a therapeutic target of β‐thalassemia.^22^ BCL11A can bind to the distal promoter regions of HbF and represses its expression.^23^, ^24^ Moreover, BCL11A is a target of Krueppel‐like factor 1 (KLF1), and inhibiting KLF1 expression is associated with repression of γ‐globin.^25^, ^26^ In erythroid cells, zinc finger and BTB domain‐containing protein 7A (ZBTB7A) also block the expression of HbF,^27^ and KLF1 directly drives ZBTB7A expression by binding to its promoter regions.^28^, ^29^ HBS1‐like translational GTPase‐MYB proto‐oncogene (MYB) also plays a critical role in regulating HbF expression.^30^, ^31^, ^32^, ^33^

MicroRNAs (miRNAs) regulate globin gene switching through post‐transcriptional mechanisms.^34^, ^35^ For example, *hsa‐miR‐15a* and *hsa‐miR‐16* target the MYB transcription factor to elevate γ‐globin expression.^36^, ^37^ Moreover, *hsa‐miR‐210*, *hsa‐miR‐30a*, and *hsa‐miR‐486‐3p* regulate γ‐globin gene expression through the post‐transcriptional regulation of BCL11A expression.^38^, ^39^, ^40^ Importantly, *let7* miRNAs have also been implicated in the developmental progression of fetal and adult human erythroblasts.^41^ In K‐562 cells, *hsa‐miR‐26b* specifically activates the transcription factor GATA1 to increase the expression of γ‐globin.^42^ With the development of sequencing technology, more differentially expressed genes, long noncoding RNAs, and miRNAs have been identified in patients with β‐thalassemia.^43^, ^44^

Because of the different expression profiles of pediatric and adult blood cells,^45^ we hypothesized that pediatric and adult β‐thalassemia may have different molecular characteristics. Differentially expressed miRNAs in adolescent or adult patients with β‐thalassemia had been reported in a previous study.^46^ However, the miRNA expression profiles in pediatric β‐thalassemia were unclear. Accordingly, in this study, we determined the miRNA expression profiles modulated in pediatric patients with β‐thalassemia. Our analysis suggested that abnormal regulation of transcriptional networks mediated by *let7* miRNAs was critical for the pathogenesis of pediatric β‐thalassemia.

## 2 MATERIALS AND METHODS

### 2.1 Study participants

This study was approved by the institutional ethics committee of our hospital (approval no. 201, 2018). 5 ml peripheral blood was collected from five pediatric patients with β‐thalassemia and five healthy controls in Fujian Maternity and Child Health Hospital, Fujian, China. The red cells were lysed using PAXgene Blood RNA Kit. The remaining mononuclear cells were used for further RNA isolation. The information and clinical conditions of the participants were also collected.

### 2.2 Total RNA isolation

Total RNA from mononuclear cells was isolated using a miRNeasy Mini Kit (Qiagen) according to the manufacturer's protocol. Briefly, mononuclear cells were lysed using lysis reagent, and 140 μl chloroform was added. The upper aqueous phase was then mixed with 100% ethanol, and the mixture was transferred to the column, washed, and eluted with RNase‐free water.

### 2.3 MicroRNA library construction and sequencing

Total RNA was used to prepare the miRNA sequencing library. After linker ligation, cDNA synthesis, and polymerase chain reaction (PCR) amplification, 135–155‐bp PCR amplification fragments were selected. The library was denatured into single‐stranded DNA, captured on an Illumina flow cell, amplified into clusters, and sequenced for 51 cycles using an Illumina NextSeq 500 sequencer (Illumina).

### 2.4 Data processing

After sequencing, Solexa Chastity software was used for quality control. The linkers were removed using Cutadapt,^47^ leaving tags with lengths greater than or equal to 15 as the trimmed reads. We used miRDeep2 software to quantify known miRNAs.^48^ Counts per million reads (CPM) were used to represent the expression levels of miRNAs. The differentially expressed miRNAs between pediatric patients with β‐thalassemia and healthy controls were determined using edgeR (version 3.32.1, http://bioconductor.org/packages/release/bioc/html/edgeR.html) in R statistics software,^49^ based on an absolute fold change greater than 1.5, *p* value less than 0.05, and CPM greater than or equal to 1.

### 2.5 Volcano plot and Venn diagram plot

Volcano plots and Venn diagrams were generated using Fancy Volcano Plot and Wonderful Venn in TBtools software (version x32_1_064, https://github.com/CJ‐Chen/TBtools), respectively.^50^

### 2.6 Heatmap presentation

Unsupervised heatmaps were generated using “pheatmap” package (version 1.0.12, https://cran.r‐project.org/web/packages/pheatmap/) in R statistics software.

### 2.7 Prediction of the target genes of miRNAs

The targets of miRNAs were predicted using miRDB (http://mirdb.org/)^51^ and TargetScan Human 7.2 (http://www.targetscan.org/vert_72/)^52^ online tools. Target genes were predicted in both miRDB and TargetScan and were selected for further analyses.

### 2.8 Biological process annotations and Kyoto Encyclopedia of Genes and Genomes (KEGG) signaling pathway analysis

The enriched biological processes and KEGG signaling pathways were determined using the Database for Annotation, Visualization, and Integrated Discovery Web site (version 6.8; https://david.ncifcrf.gov).^53^, ^54^ Statistical significance was set at *p* < 0.05.

### 2.9 Gene Expression Omnibus (GEO) data collection

The gene expression matrix from patients with β‐thalassemia and normal controls was deposited in the GSE56088 dataset from the GEO Web site (www.ncbi.nlm.nih.gov/geo). Microarray expression was analyzed using R software (version 3.5.0; https://www.r‐project.org/).

### 2.10 Analysis of the let7 miRNA‐associated transcriptional network

A network of *let7* miRNAs and predicted target genes was created using Cytoscape (http://www.cytoscape.org/).

### 2.11 Statistical analysis

Box plots were generated using GraphPad Prism software. Statistical analysis was performed using Student's *t* tests. Statistical significance was set at *p* < 0.05.

## 3 RESULTS

### 3.1 MiRNA expression profiling of pediatric β‐thalassemia

Peripheral blood samples from five children diagnosed with β‐thalassemia in our hospital and five healthy children were collected to identify differentially expressed miRNAs. The clinical characteristics of β‐thalassemia and normal individuals are shown in Table 1. The mean age of the β‐thalassemia and normal individuals was 3 years and two‐six years, respectively. Compared with normal individuals, the mean hemoglobin, red blood cell, and hematocrit in the β‐thalassemia patients were significantly lower. Moreover, the mean corpuscular hemoglobin level was also decreased in β‐thalassemia patients. However, the mean corpuscular volume and platelet count were not significantly different between the β‐thalassemia and normal cohorts.

**TABLE 1:** Clinical characteristics of patients with β‐thalassemia and normal individuals

	Control	β‐thalassemia	*p* Value
Sex (male/female)	3/2	3/2	
Age (years)	3.00 ± 1.22	2.6 ± 1.18	0.67
Hemoglobin (g/L)	124.6 ± 8.56	59 ± 5.34	<0.001
Red blood cell (10^12^/L)	4.47 ± 0.18	2.86 ± 0.49	<0.001
Hematocrit (%)	36.24 ± 1.54	21.1 ± 4.68	<0.001
Mean corpuscular volume (fl)	80.44 ± 3.05	73.92 ± 8.22	0.14
Mean corpuscular hemoglobin (pg)	27.88 ± 1.52	23.8 ± 2.87	0.02
Mean corpuscular hemoglobin concentration (g/L)	346.6 ± 18.09	302.4 ± 47.37	0.09
Platelet count (10^9^/L)	258.4 ± 84.24	275.2 ± 141.33	0.82

**FIGURE 1:**
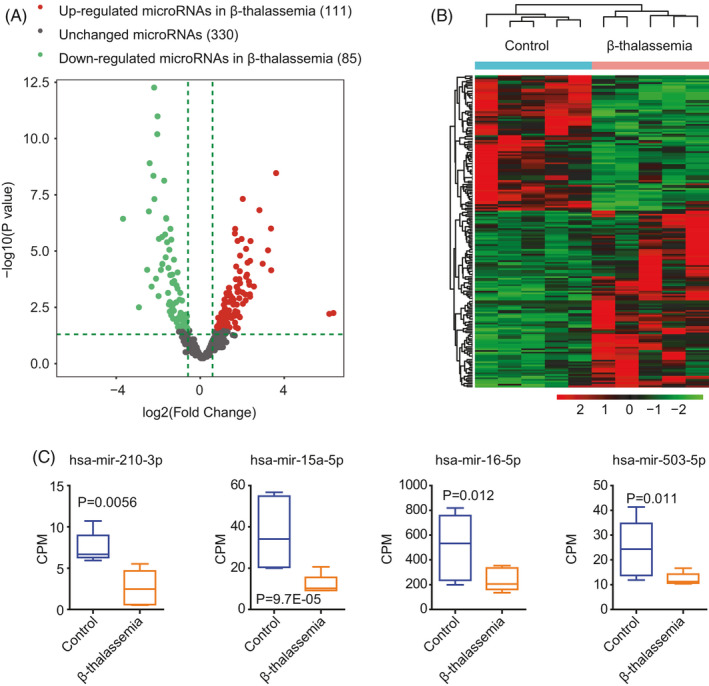
miRNA expression profiling of pediatric patients with β‐thalassemia. (A) Volcano plot showed the expression of miRNAs in pediatric patients with β‐thalassemia and normal individuals. The green dots represent downregulated miRNAs, whereas the red dots represent upregulated miRNAs in pediatric patients with β‐thalassemia. (B) The unsupervised clustering heatmap shows differentially expressed miRNAs between pediatric patients with β‐thalassemia and normal controls. Upregulated (red) and downregulated (blue) miRNAs in β‐thalassemia are showed. (C) Box plots show *hsa‐miR‐210‐3p*, *hsa‐miR‐15a‐5p*, *hsa‐miR‐16‐5p*, and *hsa‐miR‐503‐5p* expression levels in pediatric patients with β‐thalassemia and normal individuals. *P* values were generated using two‐tailed paired Student's *t* tests

Next, we performed miRNA sequencing to identify the miRNA expression profile of pediatric β‐thalassemia. In total, 530 miRNAs were identified; among these, 111 miRNAs were upregulated, whereas 85 miRNAs were downregulated in patients with β‐thalassemia. Three hundred and 330 miRNAs showed no differential expression in pediatric β‐thalassemia and normal cohorts, respectively (Figure 1A). All differentially expressed miRNAs were further illustrated using a heatmap. The results showed that these miRNAs could clearly distinguish normal individuals from patients with β‐thalassemia (Figure 1B).

Some miRNAs have been reported to be abnormally regulated in β‐thalassemia. For example, *hsa‐miR‐210* regulates γ‐globin expression through the transcription factor BCL11A,^38^ and *miR‐15a* and *miR‐16‐1* elevate γ‐globin expression through the transcription factor MYB.^36^, ^37^ Additionally, *hsa‐miR‐503* has been shown to be downregulated in patients with β‐thalassemia.^55^ Consistent with these results, we found that *hsa‐miR‐2100‐3p*, *hsa‐miR‐15a‐5p*, *hsa‐miR‐16‐5p*, and *hsa‐miR‐503‐5p* were all downregulated in pediatric patients with β‐thalassemia compared with that in normal individuals (Figure 1C). These results suggested that our miRNA sequencing data were accurate.

### 3.2 Top 10 miRNAs up‐ or downregulated in pediatric β‐thalassemia

Based on fold changes, we further evaluated the top 10 miRNAs up‐ or downregulated in pediatric patients with β‐thalassemia. Interestingly, *let7* microRNAs were particularly altered in these patients. We found that *hsa‐let‐7f‐1‐3p* and *hsa‐let‐7a‐3p* were significantly upregulated in pediatric patients with β‐thalassemia (Table 2). By contrast, *hsa‐let‐7b‐5p*, *hsa‐let‐7i‐5p*, and *hsa‐let‐7g‐5p* were significantly downregulated in these patients (Table 3). These results highlighted the abnormal regulation of *let7* microRNAs in pediatric β‐thalassemia.

**TABLE 2:** Top 10 miRNAs upregulated in pediatric β‐thalassemia

Mature ID	Fold change	*p* Value	Q value
*hsa‐miR‐483‐3p*	9.62893197	0.00012347	0.0013254
*hsa‐let‐7f‐1‐3p*	9.567670194	1.73077E‐06	4.508E‐05
*hsa‐let‐7a‐3p*	7.268324435	6.36E‐05	0.0007823
*hsa‐miR‐543*	6.532258748	2.63949E‐07	1.157E‐05
*hsa‐miR‐433‐3p*	5.450896085	0.000651725	0.0050217
*hsa‐miR‐4435*	4.868506895	6.24003E‐06	0.0001059
*hsa‐miR‐329‐3p*	4.798724188	4.72461E‐05	0.0006213
*hsa‐miR‐92b‐5p*	4.795958936	0.001835974	0.0106814
*hsa‐miR‐6747‐3p*	4.762721817	0.001442795	0.0091435
*hsa‐miR‐495‐3p*	4.695605525	0.000547849	0.0043662

**TABLE 3:** Top 10 miRNAs downregulated in pediatric β‐thalassemia

Mature ID	Fold change	*p* Value	Q value
*hsa‐miR‐4508*	0.072355279	6.38518E‐07	2.099E‐05
*hsa‐miR‐20a‐5p*	0.169841036	3.00687E‐07	1.217E‐05
*hsa‐let‐7b‐5p*	0.174221614	2.17096E‐09	2.855E‐07
*hsa‐miR‐93‐5p*	0.197344161	7.88045E‐09	5.922E‐07
*hsa‐let‐7i‐5p*	0.201976493	9.45983E‐13	4.976E‐10
*hsa‐miR‐6501‐5p*	0.203672013	8.51904E‐08	4.074E‐06
*hsa‐miR‐221‐3p*	0.224135802	1.11212E‐10	1.95E‐08
*hsa‐let‐7g‐5p*	0.226226447	1.79675E‐11	4.725E‐09
*hsa‐miR‐106a‐5p*	0.236545656	4.97221E‐06	0.0001018
*hsa‐miR‐17‐5p*	0.262349916	4.10249E‐06	8.991E‐05

Notably, *hsa‐miR‐483‐3p*, *hsa‐miR‐543*, *hsa‐miR‐433‐3p*, *hsa‐miR‐4435*, *hsa‐miR‐329‐3p*, *hsa‐miR‐92b‐5p*, *hsa‐miR‐6747‐3p*, and *hsa‐miR‐495‐3p* were upregulated (Table 2), whereas *hsa‐miR‐4508*, *hsa‐miR‐20a‐5p*, *hsa‐miR‐93‐5p*, *hsa‐miR‐6501‐5p*, *hsa‐miR‐221‐3p*, *hsa‐miR‐106a‐5p*, and *hsa‐miR‐17‐5p* were downregulated in pediatric β‐thalassemia (Table 3). Most of these miRNAs had not previously been reported to be involved in the pathology of β‐thalassemia.

### 3.3 Downregulation of let7 miRNAs in pediatric and adult patients with β‐thalassemia

**FIGURE 2:**
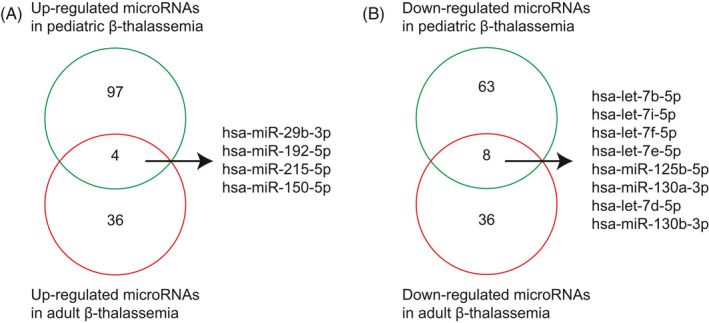
Overlapped differentially expressed miRNAs in pediatric and adult β‐thalassemia. (A) Venn diagram showing common upregulated miRNAs in pediatric and adult patients with β‐thalassemia. (B) Venn diagram showing common downregulated miRNAs in pediatric and adult patients with β‐thalassemia

Differentially expressed miRNAs in adolescent or adult patients with β‐thalassemia have been reported in a previous study.^46^ By integrating these datasets with our current results, we determined the unique and common miRNAs associated with pediatric and adult β‐thalassemia. Four miRNAs, that is, *hsa‐miR‐29b‐3p*, *hsa‐miR‐192‐5p*, *hsa‐miR‐215‐5p*, and *hsa‐miR‐150‐5p*, were upregulated in pediatric and adult patients with β‐thalassemia (Figure 2A). Five *let7* microRNAs, that is, *hsa‐let‐7b‐5p*, *hsa‐let‐7i‐5p*, *hsa‐let‐7f‐5p*, *hsa‐let‐7e‐5p*, and *hsa‐let‐7d‐5p*, were downregulated in pediatric and adult patients with β‐thalassemia (Figure 2B). Moreover, three miRNAs, that is, *hsa‐miR‐125b‐5p*, *hsa‐miR‐130a‐3p*, and *hsa‐miR‐130b‐3p*, were both altered in pediatric and adult patients with β‐thalassemia (Figure 2B). These results partially validated our analysis and again suggested that *let7* microRNAs were critical to the pathology of β‐thalassemia.

### 3.4 Transcriptional networks mediated by let7 microRNAs

**FIGURE 3:**
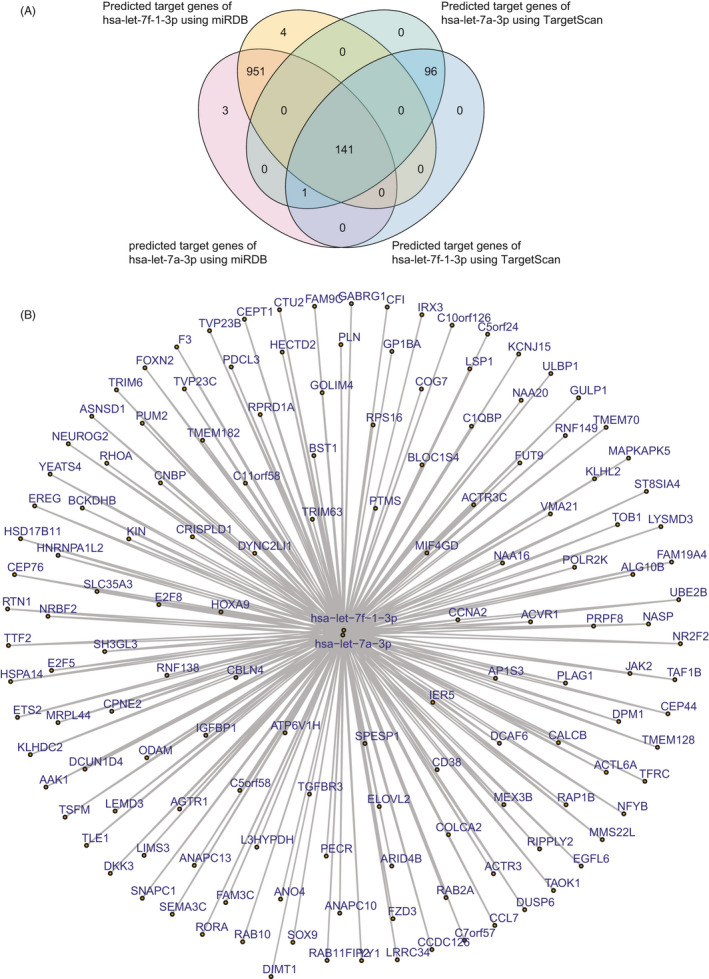
Transcriptional network mediated by *hsa‐let‐7a‐3p* and *hsa‐let‐7f‐1‐3p*. (A) Venn diagram showing common targets of *hsa‐let‐7a‐3p* and *hsa‐let‐7f‐1‐3p*, predicated by miRDB and TargetScan. (B) Transcriptional networks of *hsa‐let‐7a‐3p*, *hsa‐let‐7f‐1‐3p*, and their target genes

MiRNAs may regulate the expression of γ‐globin through post‐transcriptional silencing of target genes.^34^, ^35^ Therefore, using miRDB and TargetScan online tools, we next identified the target genes of *hsa‐let‐7a‐3p* and *hsa‐let‐7f‐1‐3p*, which were upregulated in pediatric patients with β‐thalassemia. Interestingly, we found that *hsa‐let‐7a‐3p* target genes were also predicted to be the target genes of *hsa‐let‐7f‐1‐3p*. Using miRDB, 1092 genes were predicted to be targets of *hsa‐let‐7a‐3p* and *hsa‐let‐7f‐1‐3p*. In total, 238 genes were predicted to be targets of *hsa‐let‐7a‐3p* and *hsa‐let‐7f‐1‐3p*. Overlapping the results from miRDB and TargetScan, we identified 142 genes that were targets of *hsa‐let‐7a‐3p* and *hsa‐let‐7f‐1‐3p* (Figure 3A). The connections of *hsa‐let‐7a‐3p* and *hsa‐let*‐*7f‐1‐3p* with their target genes are shown in Figure 3B.

**FIGURE 4:**
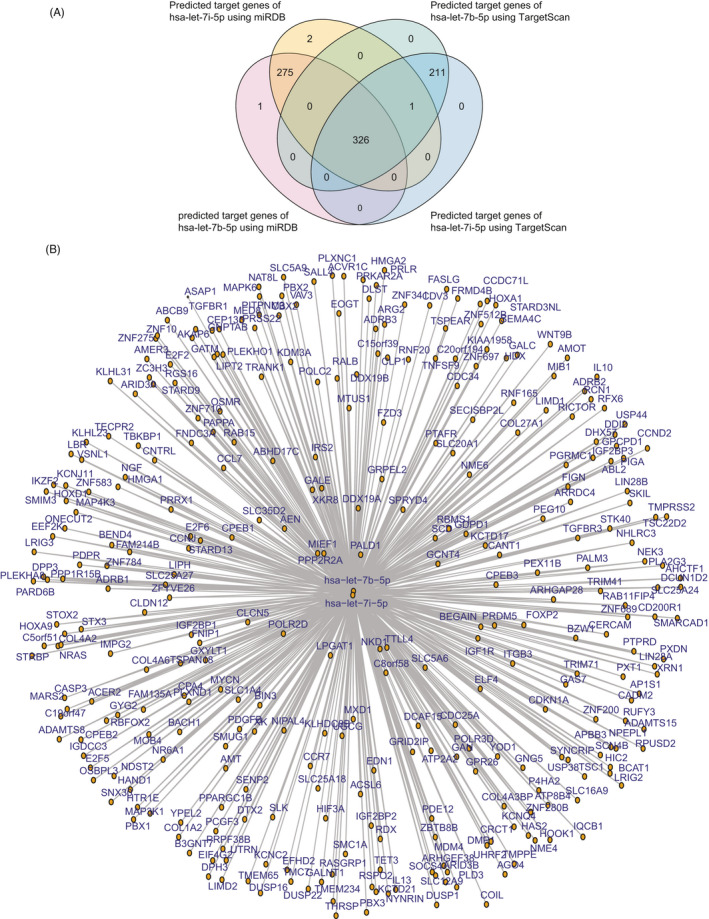
Transcriptional network mediated by *hsa‐let‐7b‐5p* and *hsa‐let‐7i‐5p*. (A) Venn diagram showing common targets of *hsa‐let‐7b‐5p* and *hsa‐let‐7i‐5p*, predicated by miRDB and TargetScan. (B) Transcriptional networks of *hsa‐let‐7b‐5p*, *hsa‐let‐7i‐5p*, and their target genes

Similarly, the target genes of *hsa‐let‐7b‐5p* and *hsa‐let‐7i‐5p*, which were downregulated in pediatric and adult patients with β‐thalassemia, were identified using the miRDB and TargetScan online tools. The target genes of *hsa‐let‐7b‐5p* and *hsa‐let‐7i‐5p* were quite similar. We identified 326 genes as targets of *hsa‐let‐7a‐3p* and *hsa‐let‐7f‐1‐3p* (Figure 4A). The connections between *hsa‐let‐7b‐5p* and *hsa‐let‐7i‐5p* and their target genes were also demonstrated (Figure 4B).

### 3.5 Biological processes and signaling pathways associated with the target genes of let7 miRNAs

**FIGURE 5:**
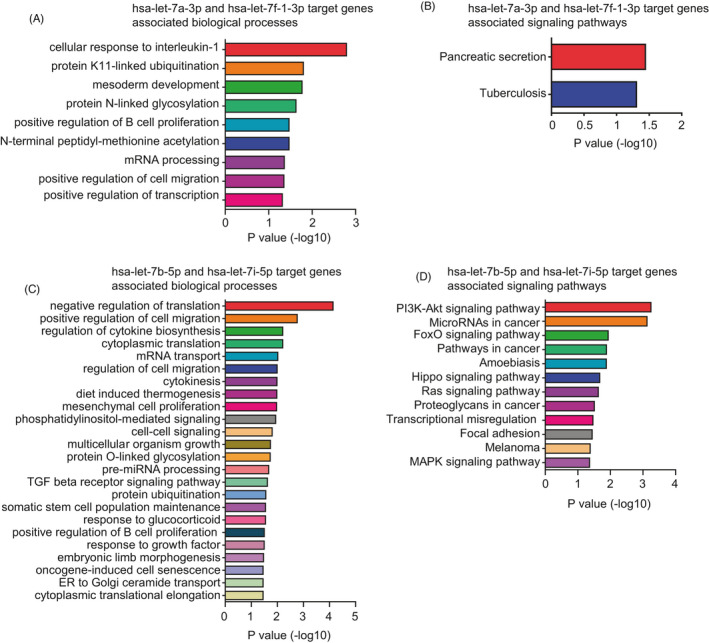
Biological processes and signaling pathways associated with target genes of *let7* miRNAs. (A) Biological processes associated with common targets of *hsa‐let‐7a‐3p* and *hsa‐let‐7f‐1‐3p*. (B) Functional pathway enrichment analysis of common targets of *hsa‐let‐7a‐3p* and *hsa‐let‐7f‐1‐3p*. (C) Biological processes associated with common targets of *hsa‐let‐7b‐5p* and *hsa‐let‐7i‐5p*. (D) Functional pathway enrichment analysis of common targets of *hsa‐let‐7b‐5p* and *hsa‐let‐7i‐5p*

Next, the biological processes and signaling pathways associated with the target genes of *hsa‐let‐7a‐3p* and *hsa‐let‐7f‐1‐3p* were determined. We found that targets of *hsa‐let‐7a‐3p* and *hsa‐let‐7f‐1‐3p* were involved in the cellular response to interleukin‐1, protein K11‐linked ubiquitination, mesoderm development biological processes (Figure 5A), pancreatic secretion, and tuberculosis signaling pathways (Figure 5B). However, how these biological processes and signaling pathways are involved in the pathology of β‐thalassemia is not clear.

The targets of *hsa‐let‐7b‐5p* and *hsa‐let‐7i‐5p* were involved in the negative regulation of translation, positive regulation of cell migration, regulation of cytokine biosynthesis, transforming growth factor (TGF) β receptor signaling pathway, and somatic stem cell population maintenance (Figure 5C). TGFβ is an important cytokine involved in cell migration^56^, ^57^ and negatively regulates erythrocyte differentiation and maturation in the early stages of erythropoiesis.^58^ In addition, somatic stem cell population maintenance is associated with changes in genes in adult β‐thalassemia.^46^

The targets of *hsa‐let‐7b‐5p* and *hsa‐let‐7i‐5p* were associated with the phosphatidylinositol 3‐kinase (PI3K)/AKT, FoxO, Hippo, and mitogen‐activated protein kinase (MAPK) signaling pathways (Figure 5D). FOXO3 is a downstream transcription factor of the PI3K/AKT signaling pathway and is also involved in the FoxO signaling pathway.^59^ Previous results have shown that the PI3K/AKT signaling pathway^60^ and FOXO3^61^, ^62^ are important regulators of erythroid maturation during erythropoiesis. The MAPK signaling pathway also participates in the development of erythropoiesis.^63^ KEGG pathway enrichment analysis identified several other pathways involved in the regulation of γ‐globin expression and the development of erythropoiesis, including the Hippo signaling pathway, RAS signaling pathway, and transcriptional dysregulation (Figure 5D).

### 3.6 Differentially expressed target genes of let7 miRNAs in β‐thalassemia

**FIGURE 6:**
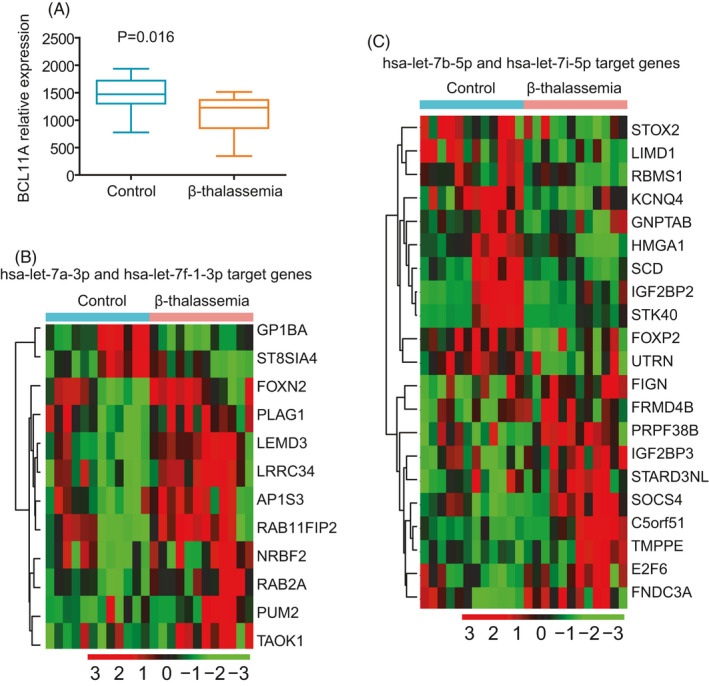
Expression levels of target genes of *let7* miRNAs in β‐thalassemia. (A) Box plots showing the expression levels of *BCL11A* in patients with β‐thalassemia and normal individuals in the GSE56088 dataset. (B) Unsupervised clustering heatmap showing the expression levels of common targets of *hsa‐let‐7a‐3p* and *hsa‐let‐7f‐1‐3p* in patients with β‐thalassemia and normal individuals in the GSE56088 dataset. (C) Expression levels of common targets of *hsa‐let‐7b‐5p* and *hsa‐let‐7i‐5p* in patients with β‐thalassemia and normal individuals

In a previous study, differentially expressed genes between patients with β‐thalassemia and healthy controls were studied; the data were deposited in the GSE56088 dataset. Using this dataset, we determined the expression of the targets of *let7* miRNAs. BLC11A is a critical transcription factor that regulates hemoglobin switching and is a target of *let7* miRNAs.^64^ First, we showed that the expression of *BLC11A* was significantly downregulated in patients with β‐thalassemia compared with that in normal individuals in the GSE56088 dataset (Figure 6A).

We also identified 12 target genes of *hsa‐let‐7a‐3p* and *hsa‐let‐7f‐1‐3p*, which were differentially expressed in patients with β‐thalassemia (Figure 6B). *GP1BA* and *ST8SIA4* were downregulated, whereas *FOXN2*, *PLAG1*, *LEMD3*, *LRRC34*, *AP1S3*, *RAB11FIP2*, *NRBF2*, *RAB2A*, *PUM2*, and *TAOK1* were upregulated in patients with β‐thalassemia. Additionally, 21 target genes of *hsa‐let‐7b‐5p* and *hsa‐let‐7i‐5p* were differentially expressed in patients with β‐thalassemia (Figure 6C). *STOX2*, *LIMD1*, *RBMS1*, *KCNQ4*, *GNPTAB*, *HMGA1*, *SCD*, *IGF2BP2*, *STK40*, *FOXP2*, and *UTRN* were downregulated, whereas *FIGN*, *FRMD4B*, *PRPF38B*, *IGF2BP3*, *STARD3NL*, *SOCS4*, *C5orf51*, *TMPPE*, *E2F6*, and FNDC3A were upregulated in patients with β‐thalassemia.

### 3.7 Hsa‐miR‐190‐5p and hsa‐miR‐1278‐5p may regulate hemoglobin switching by modulation of BCL11A

**FIGURE 7:**
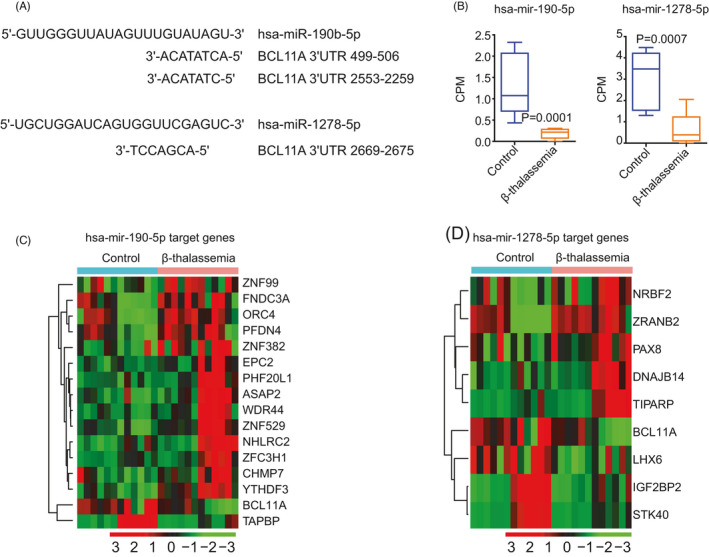
Regulatory effects of *hsa‐miR‐190‐5p* and *hsa‐miR‐1278‐5p* on hemoglobin switching via modulation of *BCL11A*. (A) Sequences of *hsa‐miR‐190‐5p*, *hsa‐miR‐1278‐5p*, and their binding sites on *BCL11A*. (B) Box plots showing *hsa‐miR‐190‐5p* and *hsa‐miR‐1278‐5p* expression levels in patients with β‐thalassemia and normal individuals. (C) Unsupervised clustering heatmap showing the expression levels of targets of *hsa‐miR‐190‐5p* in patients with β‐thalassemia and normal individuals. (D) Expression levels of targets of *hsa‐miR‐1278‐5p* in patients with β‐thalassemia and normal individuals

BLC11A is a critical transcription factor that regulates hemoglobin switching.^19^, ^20^, ^21^ Finally, we attempted to identify novel microRNAs regulating hemoglobin switching via modulation of *BCL11A* expression. Using miRDB, we found that two miRNAs, that is, *hsa‐miR‐190‐5p* and *hsa‐miR‐1278‐5p*, targeted *BCL11A*. In particular, *hsa‐miR‐190‐5p* was perfectly matched to the two 3′ untranslated regions (UTRs) of *BCL11A* (Figure 7A). In addition, *hsa‐miR‐1278‐5p* targeted the 3′ UTRs of *BCL11A* (Figure 7A). Moreover, *hsa‐miR‐190‐5p* and *hsa‐miR‐1278‐5p* were both downregulated in patients with β‐thalassemia compared with those in normal controls (Figure 7B).

We further identified the target genes of *hsa‐miR‐190‐5p* and *hsa‐miR‐1278‐5p*. In addition to *BCL11A*, *hsa‐miR‐190‐5p* also targeted *ZNF99*, *FNDC3A*, *ORC4*, *PFDN4*, *ZNF382*, *EPC2*, *PHF20L1*, *ASAP2*, *WDR44*, *ZNF529*, *NHLRC2*, *ZFC3H1*, *CHMP7*, *YTHDF3*, and *TAPBP* genes (Figure 7B). Furthermore, *hsa‐miR‐190‐5p* also targeted *NRBF2*, *ZRANB2*, *PAX8*, *DNAJB14*, *TIPARP*, *LHX6*, *IGF2BP2*, and *STK40* (Figure 7C). Interestingly, *IGF2BP2* and *STK40* were also target genes of *hsa‐let‐7a‐3p* and *hsa‐let‐7f‐1‐3p* (Figure 6B). However, the functions of *hsa‐miR‐190‐5p* and *hsa‐miR‐1278‐5p* in hemoglobin switching and β‐thalassemia need to be studied in greater detail.

## 4 DISCUSSION

β‐Thalassemia is a heterogeneous disease, and the clinical manifestations of β‐thalassemia in pediatric and adult patients may be different.^65^, ^66^ Because of the high hematopoietic stem cell repopulating capacity in children and the impaired functions of the bone marrow niche during aging, pediatric patients with β‐thalassemia have a superior therapeutic response to hematopoietic stem cell gene therapy than adult patients with β‐thalassemia.^67^ Therefore, pediatric and adult β‐thalassemia may have different molecular characteristics. In this study, we showed that the miRNAs associated with pediatric and adult β‐thalassemia were quite different. Only four miRNAs were upregulated, and eight miRNAs were downregulated in both pediatric and adult patients with β‐thalassemia. These differences were partially due to the different cohorts and approaches; however, we also revealed that five *let7* miRNAs, that is, *hsa‐let‐7b‐5p*, *hsa‐let‐7i‐5p*, *hsa‐let‐7f‐5p*, *hsa‐let‐7e‐5p*, and *hsa‐let‐7d‐5p*, may be involved in the reactivation of γ‐globin expression and HbF synthesis in pediatric and adult patients with β‐thalassemia.

Consistent with these observations, reports have shown that the *LIN28B*/*let7* axis directly regulates BCL11A expression to promote hemoglobin switching.^68^, ^69^ Targeted inhibition of *hsa‐let‐7a* and *hsa‐let‐7b* reactivated the expression of HbF in erythroid cells.^41^ However, we showed that *hsa‐let‐7a* was upregulated in pediatric β‐thalassemia, whereas *hsa‐let‐7b* was downregulated in pediatric β‐thalassemia. The functions of *hsa‐let‐7i‐5p*, *hsa‐let‐7f‐5p*, *hsa‐let‐7e‐5p*, and *hsa‐let‐7d‐5p* in the regulation of γ‐globin expression or HbF synthesis in erythroid cells have not been reported. Furthermore, we identified 21 target genes of *hsa‐let‐7b‐5p* and *hsa‐let‐7i‐5p*, which were differentially expressed in patients with β‐thalassemia. We also found that the target genes of *hsa‐let‐7b‐5p* and *hsa‐let‐7i‐5p* were associated with the PI3K/AKT, FoxO, Hippo, and MAPK signaling pathways. How those pathways involved in the pathology of β‐thalassemia should be further studied.

BCL11A,^19^, ^20^, ^21^ KLF1,^29^ and MYB^31^, ^32^ are transcription factors that play important roles in hemoglobin switching. *Hsa‐miR‐210* and *hsa‐let‐7b‐5p* regulate γ‐globin expression through BCL11A.^38^ Additionally, *miR‐15a* and *miR‐16‐1* elevate γ‐globin expression through the transcription factor MYB.^36^, ^37^ Our results showed that *hsa‐miR‐210*, *hsa‐let‐7b‐5p*, *miR‐15a*, and *miR‐16‐1* were all downregulated in β‐thalassemia. We believe that our data could help to identify more miRNAs associated with the BCL11A transcription factor. Indeed, our findings showed that two miRNAs, that is, *hsa‐miR‐190‐5p* and *hsa‐miR‐1278‐5p*, may regulate hemoglobin switching by targeting *BCL11A*. However, the functions of these miRNAs should be studied further.

To the best of our knowledge, this is the first study to identify differentially expressed miRNAs, particularly in pediatric β‐thalassemia. Our results suggest that *let7* miRNAs and their target genes are abnormally dysregulated in pediatric β‐thalassemia. However, there were some limitations to the integrated analysis of the different datasets. Because of differences in cohorts and approaches, our analysis could not fully reveal the miRNA profiles associated with pediatric and adult β‐thalassemia. In addition, identification of differentially expressed miRNA target genes in patients with β‐thalassemia using the GSE56088 dataset may also have some bias. In our subsequent studies, we will collect a large cohort of β‐thalassemia cases comprising patients of different ages and perform miRNA sequencing and mRNA sequencing simultaneously. Additionally, the functions of *hsa‐let‐7i‐5p*, *hsa‐let‐7f‐5p*, *hsa‐let‐7e‐5p*, and *hsa‐let‐7d‐5p* in the regulation of γ‐globin expression will be studied in greater detail.

## CONFLICT OF INTEREST

The authors declare that they have no conflicts of interest.

## AUTHOR CONTRIBUTIONS

HW performed the data analysis and wrote the manuscript. MH collected the blood samples. SY, YL, and YH helped with the collection of blood samples. HL and LP designed the study and supervised the work.

## Data Availability

The data generated during the current study are available from the corresponding author upon reasonable request.
